# Integrative Analysis of the Expression of SIGLEC Family Members in Lung Adenocarcinoma via Data Mining

**DOI:** 10.3389/fonc.2021.608113

**Published:** 2021-03-16

**Authors:** Haiyan Zhang, Yongfei Xie, Zhi Hu, Hong Yu, Xiang Xie, Yingchun Ye, Wenfeng Xu, Siji Nian, Qing Yuan

**Affiliations:** ^1^Public Experimental Technology Center, The School of Basic Medical Science, Southwest Medical University, Luzhou, China; ^2^Life Sciences School, Anhui Agricultural University, Hefei, China; ^3^Department of Thoracic Surgery, The Affiliated Hospital of Southwest Medical University, Luzhou, China

**Keywords:** lung adenocarcinoma, bioinformatics, SIGLEC, survival, immunotherapy

## Abstract

**Background:** Sialic acid-binding immunoglobulin-type lectin (SIGLEC) family members are involved in regulating immune-cell activation, proliferation, and apoptosis, and they play an important role in tumor development. However, their expression and correlation with immune molecules in lung adenocarcinoma (LUAD) remain unclear.

**Methods:** We utilized Gene Expression Profiling Interactive Analysis, Kaplan–Meier analysis, the limma package in R/Bioconductor, the University of California Santa Cruz Cancer Genome Browser, cBioPortal, STRING, Cytoscape, DAVID, and the Tumor Immune Estimation Resource for gene and protein profiling and analyses.

**Results:** The results showed that SIGLEC10 and SIGLEC15 levels were upregulated in LUAD, whereas SIGLEC1, CD22 (SIGLEC2), CD33, myelin-associated glycoprotein (SIGLEC4), SIGLEC5, SIGLEC6, SIGLEC7, SIGLEC8, SIGLEC11, and SIGLEC14 levels were significantly downregulated, with their low expression associated with poor overall survival. Moreover, we observed high SIGLEC-mutation rates (22%) in LUAD patients, with SIGLEC functions determined as primarily involved in regulating the immune response, signal transduction, inflammatory response, and cell adhesion. Furthermore, we found that SIGLEC expression was significantly correlated with immune-cell infiltration, especially macrophages, neutrophils, and dendritic cells, and highly associated with immune molecules such as CD80, CD86, CD28, B-cell-activating factor, programmed cell death 1 ligand 2, and colony stimulating factor 1 receptor.

**Conclusion:** These results provide insight into the potential molecular mechanism associated with SIGLEC-related development of LUAD, as well as clues for screening biomarkers and therapeutic targets.

## Introduction

High cancer rates worldwide continue to attract the attention of both scientists and medical workers. In most countries, the incidence and mortality rates of lung cancer occupies the first or second position in terms of malignancy (1). Approximately 85% of lung cancer patients are diagnosed with non-small cell lung cancer (NSCLC), of which lung adenocarcinoma (LUAD) is the most common subtype, accounting for about 40% of primary lung tumors (2). Additionally, LUAD is the most common form of lung cancer in women, patients with no history of smoking, and people aged <40 years (3). LUADs usually originate in peripheral lung tissue and can maintain localization there for extended periods before symptoms appear, the more generalized early versions of which (e.g., fatigue, subtle shortness of breath, or upper back, and chest pain) can be missed or attributed to other causes.

Targeted therapies inhibit the growth of cancer cells by blocking the activity of specific oncogenic signaling molecules (4). Some genetic alterations observed in LUAD include gene mutations in *epidermal growth factor receptor, BRAF*, and *human epidermal growth factor receptor 2*, gene fusions, and rearrangements in ALK, *ROS1*, or *RET* (5–9). Drugs, such as afatinib, alectinib, ceritinib, crizotinib, erlotinib, and gefitinib, have been approved by the Food and Drug Administration for clinical use (10), and immunotherapy has rapidly grown as a major modality for LUAD treatment (11). Binding of cytotoxic T lymphocyte-associated antigen 4 (CTLA-4) to B7-1 (CD80) and B7-2 (CD86) produces an inhibitory signal that limits the production of interleukin (IL)-2 and inhibits the proliferation and activation of T cells. The CTLA-4 antibody ipilimumab works to turn off this inhibitory mechanism or “release the brakes” in order to allow T cells to target and kill cancer cells (12, 13). Similar to CTLA-4, binding of programmed death-1 (PD-1) by its ligand (PD-L1), which is overexpressed on the surface of some cancer cells, releases signals that inhibit T cell activation and promote tumor-cell evasion (14, 15). Monoclonal antibodies, such as pembroliz, nivolumab, and atezolizumab, target PD-1 or PD-L1 to help boost the immune response to attack and destroy cancer cells (16). However, although significant advances in lung cancer treatment have been made, the 5-year survival rate for LUAD remains low. Therefore, continued investigation of potential LUAD-related genes and identification of their mechanisms is necessary (17).

Sialic acid-binding immunoglobulin-type lectins (SIGLECs) are a family of immunomodulatory receptors and a subset of I-type lectins that can recognize sialic acid sugar-carrying glycans (sialoglycans) aberrantly expressed on many tumor cells (18). SIGLECs are characterized into two distinct groups: the first and highly conserved group comprises sialoadhesin (SIGLEC1; CD169), CD22 (SIGLEC2), myelin-associated glycoprotein (MAG; SIGLEC4), and SIGLEC15; and the second, rapidly evolving group comprises CD33-related SIGLECs, including SIGLEC3/5/6/7/8/9/10/11/14/16 (19, 20). SIGLECs regulate immune-cell signaling and are involved in pathogen recognition, modulation of immune responses, and intercellular interactions (21, 22). SIGLEC1 is a macrophage-restricted cell surface receptor that has been shown to contribute to sialylated pathogen uptake, lymphocyte proliferation and antigen presentation. CD22 inhibits B cell receptor (BCR) signaling and regulates toll-like receptor (TLR) signaling and the survival of B cells. CD33, a marker of myeloid cells, plays a role in myeloid differentiation and dendritic cell maturation. MAG binds to complex gangliosides (GD1a and GT1b) to inhibit axon regeneration and neurite outgrowth. It has been shown that a paired receptor system in the SIGLEC family has implications for regulation of host immunity. SIGLEC5/SIGLEC14 and SIGLEC11/SIGLEC16 represent such paired receptors, which contain similar extracellular domains and play a role in mediating host–pathogen interactions (19). SIGLEC6 is a leptin-binding protein that has functional consequences on the aberrant proliferation, apoptosis and invasion of BeWO cells (23). SIGLEC7 is the first Siglec receptor observed on human natural killer (NK) cells, SIGLEC7/SIGLEC9 ligands shield malignant cells from NK cell attack. Expression of SIGLEC7 in multiple sclerosis (MS) patients correlates with clinical course, suggesting it as a potential biomarker of acute disease activity in MS (24). Moreover, SIGLEC9 is overexpressed on tumor-associated T cells and shifts macrophages toward tumor-promoting behaviors, suggesting that targeting SIGLEC9-related pathways might improve the antitumor response (25). SIGLEC8 has been identified as a target for the treatment of eosinophil disorders. SIGLEC10 is a suppressor receptor expressed on the surface of T cells, which triggers immunosuppression by blocking the activation of T cell receptor. SIGLEC15, as a critical immune suppressor, suppresses T-cell activation and promotes the survival and differentiation of suppressor myeloid cells. SIGLEC15 not only plays a biological role in osteoclast differentiation but also in microbial infection and tumor microenvironment (26, 27). Furthermore, SIGLECs are abnormally expressed in a variety of malignancies, including hepatocellular carcinoma, endometrial cancer, bladder cancer, and colon cancer (26), and CD33 (28) and CD22 were identified as immunotherapeutic targets for acute myeloid leukemia and non-Hodgkin's lymphoma, respectively (29, 30). A recent study indicated that sialoglycan–SIGLEC interactions in the tumor microenvironment (TME) suppress effector immune-cell activity and modulate myeloid-cell functions, thereby contributing to tumor immune evasion and sustained tumor growth (31, 32). To our knowledge, however, there are no comprehensive reports of SIGLEC-related expression and its correlation with immune molecules in LUAD.

In this study, we comprehensively analyzed the expression of SIGLEC family members in LUAD and its relationship with patient prognosis, immune-molecule expression, and infiltrating levels of immune cells in order to expand the knowledge of SIGLEC-related regulation and function, and to promote improvements in LUAD diagnosis, treatment, and prognosis.

## Materials and Methods

### Gene Expression Profiling Interactive Analysis Database

SIGLEC-expression data from 23 malignant tumors were obtained from the GEPIA database (http://gepia.cancer-pku.cn/), which is an interactive web application for gene-expression analysis based on RNA-sequencing (RNA-seq) data from The Cancer Genome Atlas (TCGA) and Genotype-Tissue Expression databases (33). Gene-expression levels were presented using the log_2_ (TPM + 1) scale.

### University of California Santa Cruz Xena Repository

LUAD RNA-seq data were downloaded from the UCSC Cancer Genome Browser (https://genome.ucsc.edu/) (34), which contained 586 samples, including 59 normal samples and 526 tumor samples. Differential-expression analysis was performed using the R package limma (35), and differentially expressed genes were screened with according to a false-discovery rate (FDR) <0.05 and a |log_2_ (fold-change)| > 1.

### Kaplan–Meier Analysis

The correlation between individual SIGLEC family member expression and LUAD patient overall survival (OS) was analyzed using a Kaplan-Meier plotter database (https://kmplot.com/analysis/). *P*-values were calculated using the log-rank test.

### cBioPortal Analysis

cBioPortal (https://www.cbioportal.org/) is an open resource for exploring, analyzing, and visualizing multidimensional cancer genomics data. Data from > 5,000 tumor samples from 20 cancer studies are currently accessible (36, 37). Genetic alteration and co-expression of SIGLECs were analyzed via public LUAD datasets.

### Protein–Protein Interaction-Network Construction

We used Venny software (v.2.1; https://bioinfogp.cnb.csic.es/tools/venny/) to highlight which differentially expressed SIGLEC-related genes. The STRING protein interaction database (38) and Cytoscape software (39) were used to analyze and reconstruct protein-interaction networks related to SIGLECs, with the top 10 hub genes analyzed by the Cytoscape plugin cytoHubba.

### Gene Ontology and Kyoto Encyclopedia of Genes and Genomes Analysis

We used the Database for Annotation, Visualization and Integrated Discovery (DAVID) bioinformatics resources version 6.8 (40) to perform functional GO and KEGG pathway enrichment analyses of genes related to SIGLEC expression. GO terms and KEGG pathways, and *p* < 0.05 were considered significantly enriched.

### Tumor Immune Estimation Resource Analysis

The correlation of SIGLECs with immune-cell infiltration in LUAD was analyzed by TIMER (41), which is a web-based platform that provides the abundance of six tumor-infiltrating immune subsets (B cells, CD4^+^ T cells, CD8^+^ T cells, neutrophils, macrophages, and dendritic cells) based on data from TCGA. Associations between SIGLEC expression and immune molecules were estimated by Spearman's correlation coefficient.

## Results

### SIGLECs Expression in Different Tumor Types

**Figure 1:**
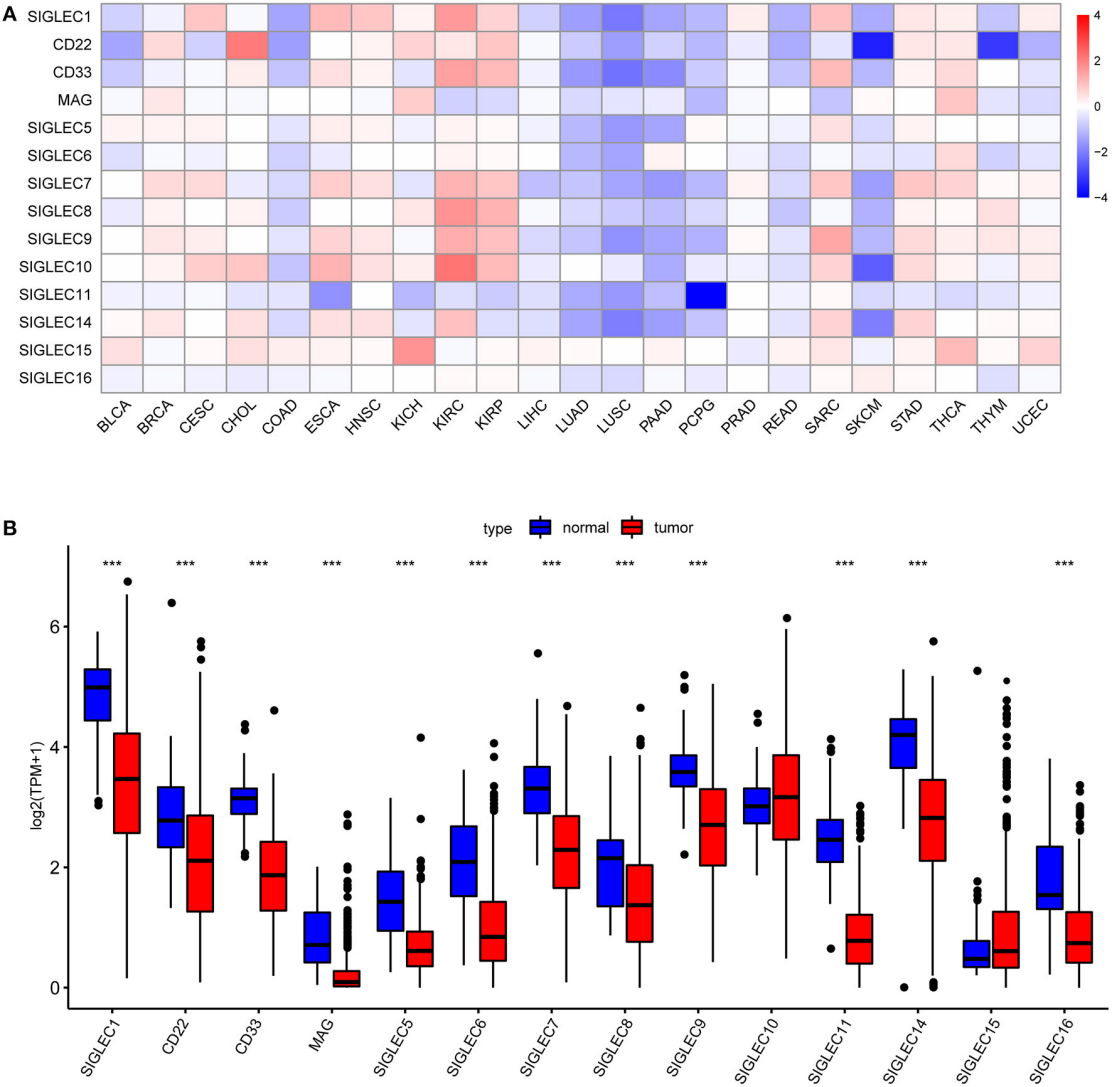
*SIGLEC* expression in different tumor types. **(A)** Increased or decreased SIGLEC expression in 23 cancer types according to comparison with normal tissues from TCGA data. Filled color represents expression fold change using gradient color from blue to red. Blue represents down-regulation, and red represents up-regulation. **(B)** Boxplots showing *SIGLEC* expression following log_2_ (TPM+1) normalization of data for normal tissues and LUAD tissues using TCGA RNA-seq data ***p < 0.001.

First, we analyzed SIGLECs expression in various tumors via the GEPIA platform. To determine aberrant SIGLECs expression, we generated a heatmap to present the log fold change in tumor tissues relative to normal tissues. The results showed that CD22 levels was significantly upregulated in cholangiocarcinoma (CHOL) and kidney chromophobe (KICH) cancer, and that SIGLEC1, CD22, CD33, SIGLEC7, SIGLEC8, SIGLEC9, and SIGLEC10 levels were upregulated in kidney renal clear cell carcinoma (KIRC) and kidney renal papillary cell carcinoma (KIRP) relative to levels in normal tissue. Notably, almost all SIGLEC members were downregulated in NSCLCs, such as LUAD and lung squamous cell carcinoma (LUSC) (Figure 1A).

We then focused on SIGLECs expression in LUAD as the most common histological type of lung cancer. Analysis of a LUAD-specific RNA-seq dataset revealed results consistent with previously analyzed data, and that mRNA levels of *SIGLEC1, CD22, CD33, MAG, SIGLEC5, SIGLEC6, SIGLEC7, SIGLEC8, SIGLEC9, SIGLEC11, SIGLEC14*, and *SIGLEC16* were downregulated in LUAD tumor tissues relative to normal tissues (*p* < 0.001) (Figure 1B). *SIGLEC10* and *SIGLEC15* expression was also upregulated in tumor tissues, although with no significant difference relative to normal tissues.

### SIGLECs Are Associated With LUAD Prognosis

**Figure 2:**
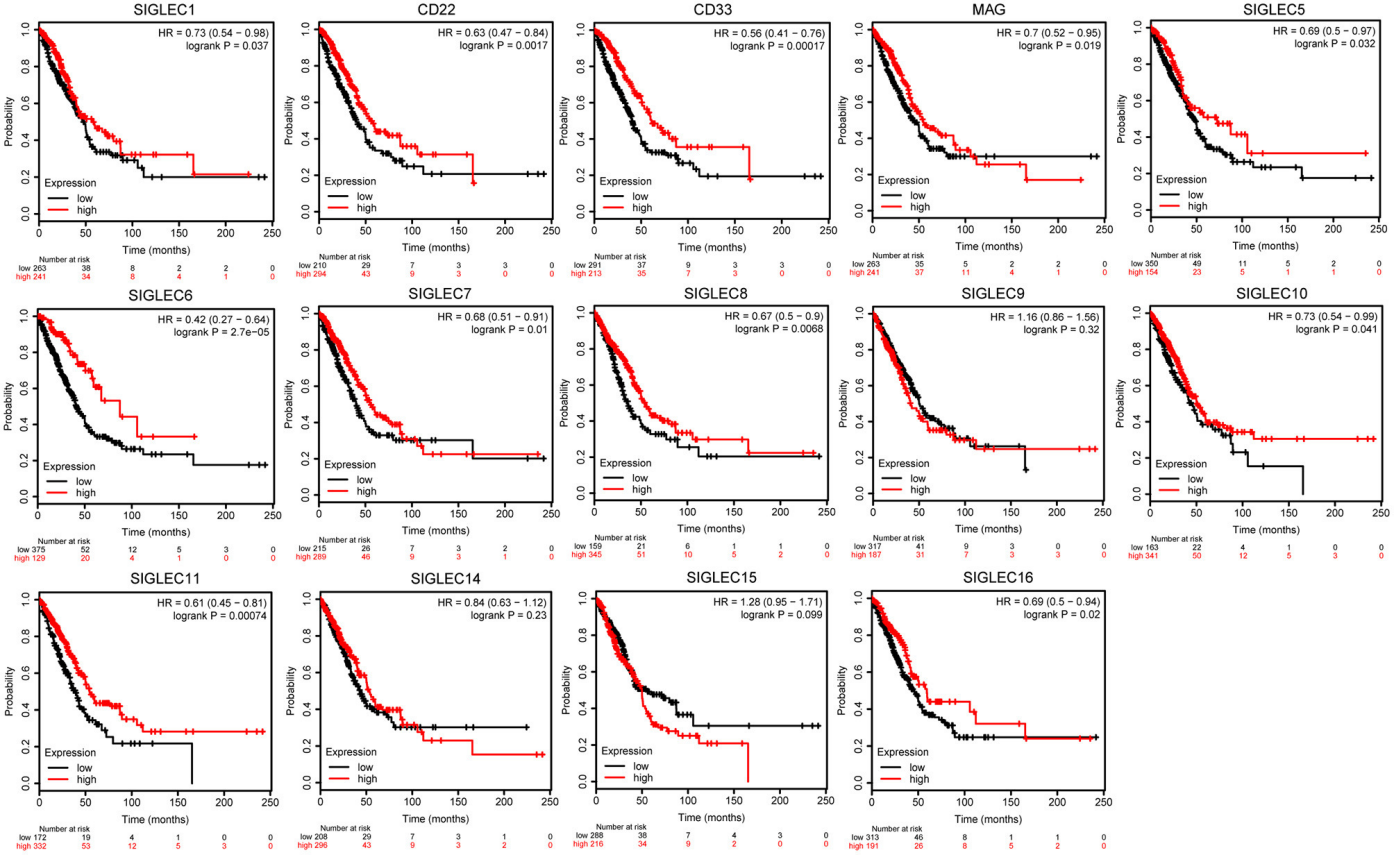
Kaplan–Meier analysis showing correlations between *SIGLEC* expression LUAD patient OS. Decreased *SIGLEC*1/2/3/4/5/6/7/8/11/16 mRNA levels were significantly associated with poor OS (*p* < 0.05). *SIGLEC*9/14/15 did not show significant prognostic values.

To investigate the effect *SIGLEC* mRNA expression on LUAD prognosis, we performed Kaplan–Meier analysis. We found that decreased levels of *SIGLEC1* [hazard ratio (HR) = 0.73, 95% confidence interval (CI): 0.54–0.98; *p* = 0.037], *CD22* [HR = 0.63, 95% CI: 0.47–0.84; *p* = 0.0017], *CD33* [HR = 0.56, 95% CI: 0.41–0.76; *p* = 0.00017], *MAG* [HR = 0.7, 95% CI: 0.52–0.95; *p* = 0.019], *SIGLEC5* [HR = 0.69, 95% CI: 0.5–0.97; *p* = 0.032], *SIGLEC6* [HR = 0.42, 95% CI: 0.27–0.64; *p* = 2.7e−05], *SIGLEC7* [HR = 0.68, 95% CI: 0.51–0.91; *p* = 0.01], *SIGLEC8* [HR = 0.67, 95% CI: 0.5–0.9; *p* = 0.037]; *SIGLEC10* [HR = 0.73, 95% CI: 0.54–0.99; *p* = 0.041], *SIGLEC11* [HR = 0.61, 95% CI: 0.45–0.81; *p* = 0.00074], and *SIGLEC16* [HR = 0.69, 95% CI: 0.5–0.94; *p* = 0.02] were associated with worse OS in LUAD patients, whereas *SIGLEC9, SIGLEC14*, and *SIGLEC15* expression were not correlated with OS (*p* > 0.05) (Figure 2).

### cBioPortal Analysis

**Figure 3:**
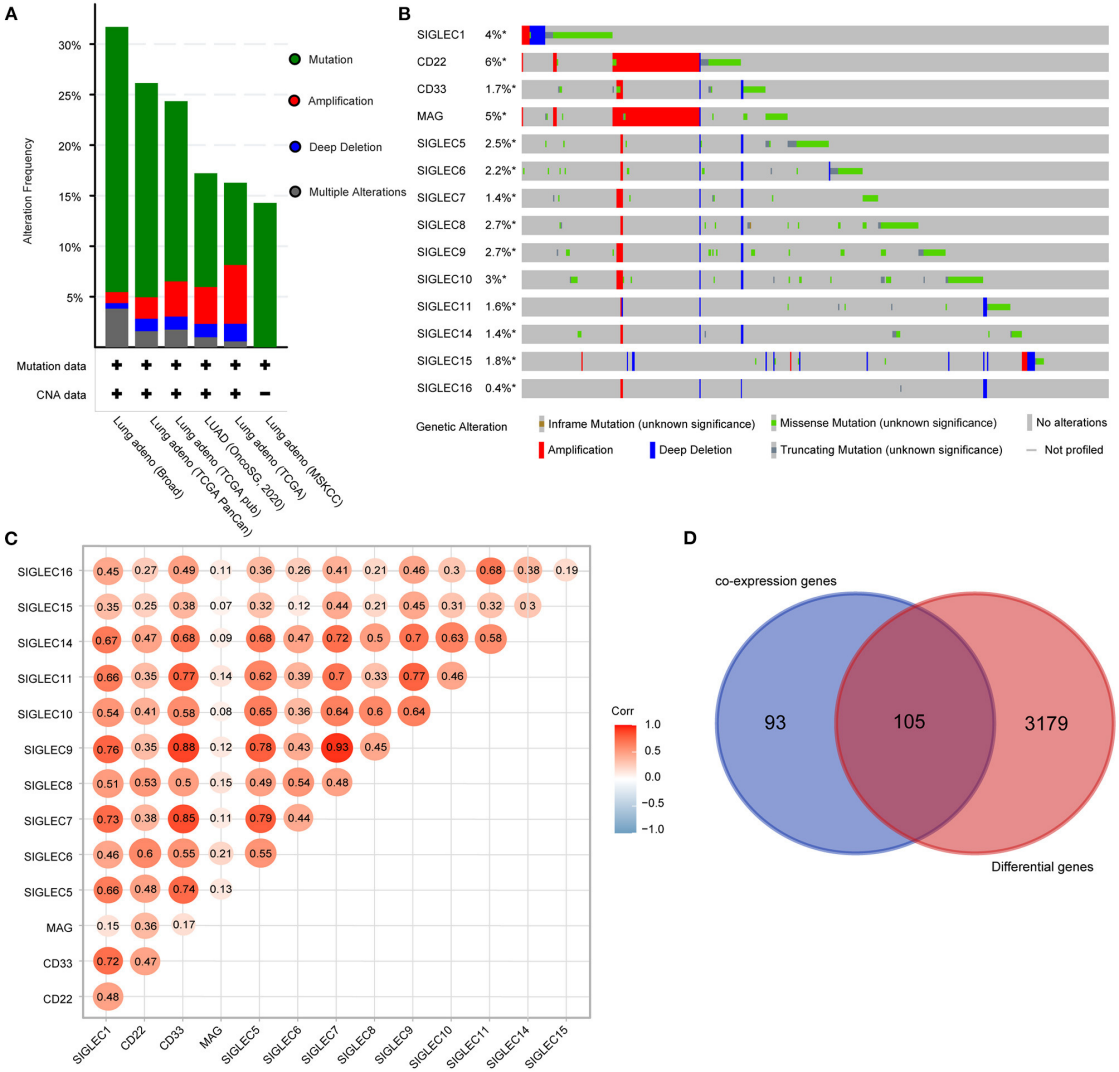
*SIGLEC* expression and alterations in LUAD. **(A)** Genetic alterations of *SIGLECs* in six TCGA datasets for LUAD. **(B)** Oncoprint displaying the distribution and proportion of samples with *SIGLEC* alterations. **(C)** Correlations between the expression of each SIGLEC family member in LUAD. **(D)** Venn diagram showing genes associated with *SIGLEC* expression and differentially expressed in LUAD.

We then analyzed the genetic alterations of *SIGLECs* based on mutation and copy number alteration data from different LUAD studies. A total of 1,832 samples were obtained from six TCGA studies, among which 403 samples (22%) involved genetic alterations of *SIGLECs* [290 (15.83%) mutations, 63 (3.44%) amplifications, 24 (1.31%) deep deletions, and 26 (1.42%) multiple alterations] (Figure 3A).

Analysis of genetic alteration frequency and types for the SIGLECs and according to tumor type (Figure 3B) showed that the alteration frequency ranged from 0.4 to 6%, with *CD22* having the largest percentage change, followed by *MAG*. Additionally, amplifications occurred in *CD22* and *MAG*, with mutations were the most common features and included truncation, missense, and in-frame mutations. Moreover, we found deep deletions in all *SIGLECs*.

Using the LUAD dataset from TCGA (*n* = 586), we performed gene co-expression analysis to determine correlations between SIGLEC family members. The results showed significant positive correlations between *SIGLEC1, CD33, SIGLEC5, SIGLEC7, SIGLEC9, SIGLEC11*, and *SIGLEC1* expression. Additionally, expression of SIGLEC10 was strongly positively correlated with that of *SIGLEC5, SIGLEC7, SIGLEC8, SIGLEC9*, and *SIGLEC14* expression (Figure 3C), whereas no correlation between *MAG* and other SIGLEC family members were observed, except for *CD22* and *SIGLEC6*.

We then evaluated other genes co-expressed with *SIGLECs*, focusing on the top 30 genes of each SIGELC, with deletion of duplicates (*n* = 198 genes). Following screening of the 3,284 differentially expressed LUAD genes, we identified 105 overlapping genes related to *SIGLEC* expression and differentially expressed in LUAD (Figure 3D).

### PPI-Network Construction

**Figure 4:**
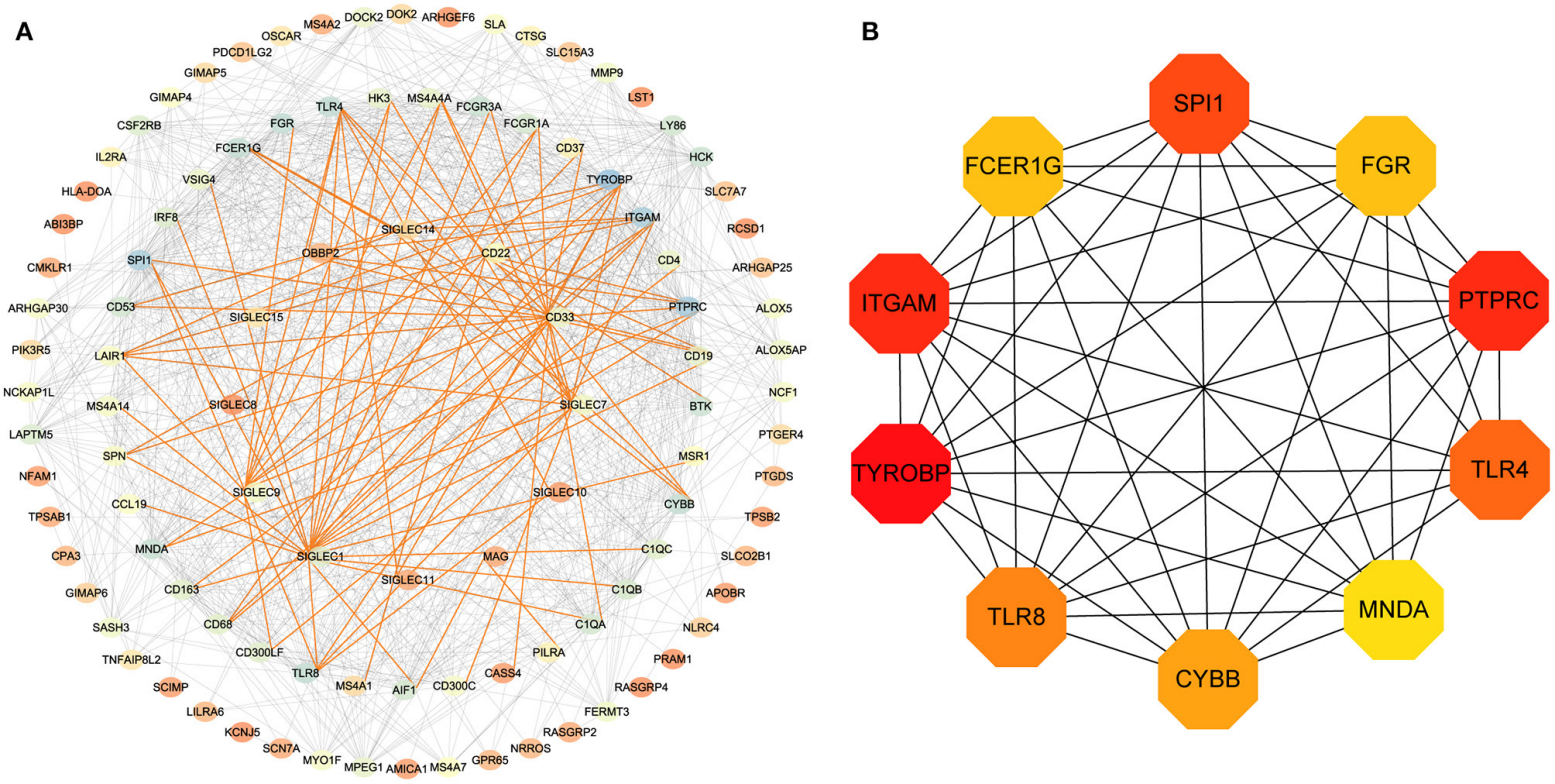
PPI-network analysis. **(A)** Network of proteins encoded by differentially expressed genes in LUAD and related to *SIGLEC* expression. The gradient of the node color from red to blue represents increases in the number of edges. The red lines represent the direct interaction between SIGLECs and differential genes. **(B)** According to the connectivity of the network, 10 genes with the highest degrees of connectivity were selected as hub genes.

To assess potential relationship between the identified genes, we generated a PPI network. As shown in Figure 4A, SIGLEC6 and SIGLEC16 did not interact with other genes. We subsequently identified 10 hub genes (Figure 4B).

### GO and KEGG Analyses

**Figure 5:**
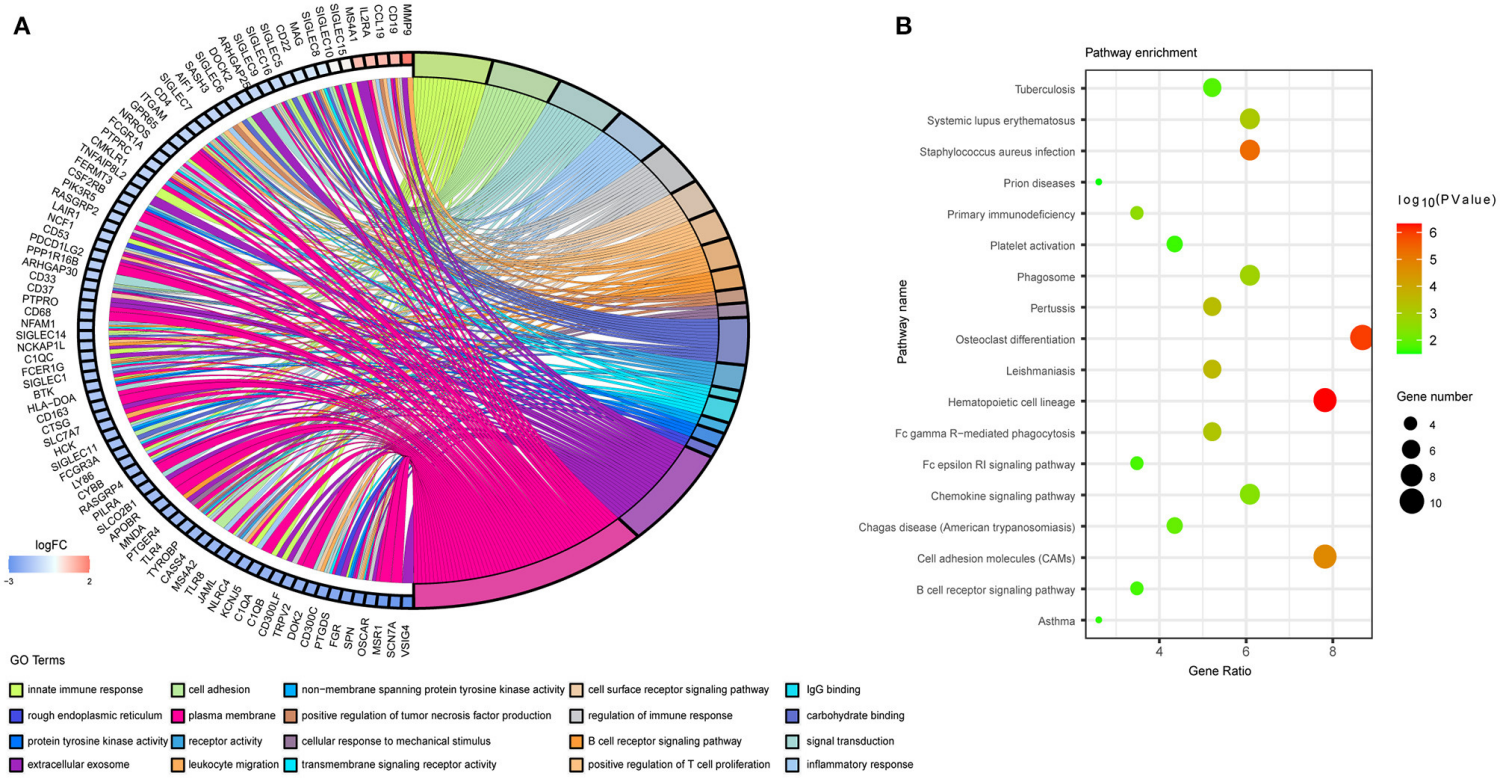
Functional annotation of the overlapping genes extracted in the Venn diagram. **(A)** Chord plot displaying the top 20 significant GO terms and their genes. FC, fold change. **(B)** Bubble plot of KEGG pathway enrichment analyses.

We then performed DAVID analysis of proteins in the PPI network for functional and pathway enrichment. We selected 20 GO terms (Figure 5A), which included cell adhesion, inflammatory response, cell-surface receptor-signaling pathway, B cell receptor-signaling pathway, signal transduction, cellular response to mechanical stimulus, positive regulation of tumor necrosis factor (TNF) production, and positive regulation of T cell proliferation among biological processes significantly associated with LUAD tumorigenesis and progression. Cellular component (CC) analysis indicated enrichment in integral components of the membrane, rough endoplasmic reticulum, extracellular exosome, and immunological synapse. Areas associated with molecular function (MF) were enriched for carbohydrate binding, receptor activity, transmembrane signaling-receptor activity, integrin binding, and non-membrane-spanning protein tyrosine kinase activity. Moreover, KEGG pathway analysis identified osteoclast differentiation, hematopoietic cell lineage, cell-adhesion molecules, the phagosome, primary immunodeficiency, Fc gamma R-mediated phagocytosis, complement and coagulation cascades, tuberculosis, the chemokine-signaling pathway, and asthma as enriched pathways (Figure 5B).

### SIGLECs Correlation With Immune-Cell Infiltration and -Molecule Levels, Respectively

**Figure 6:**
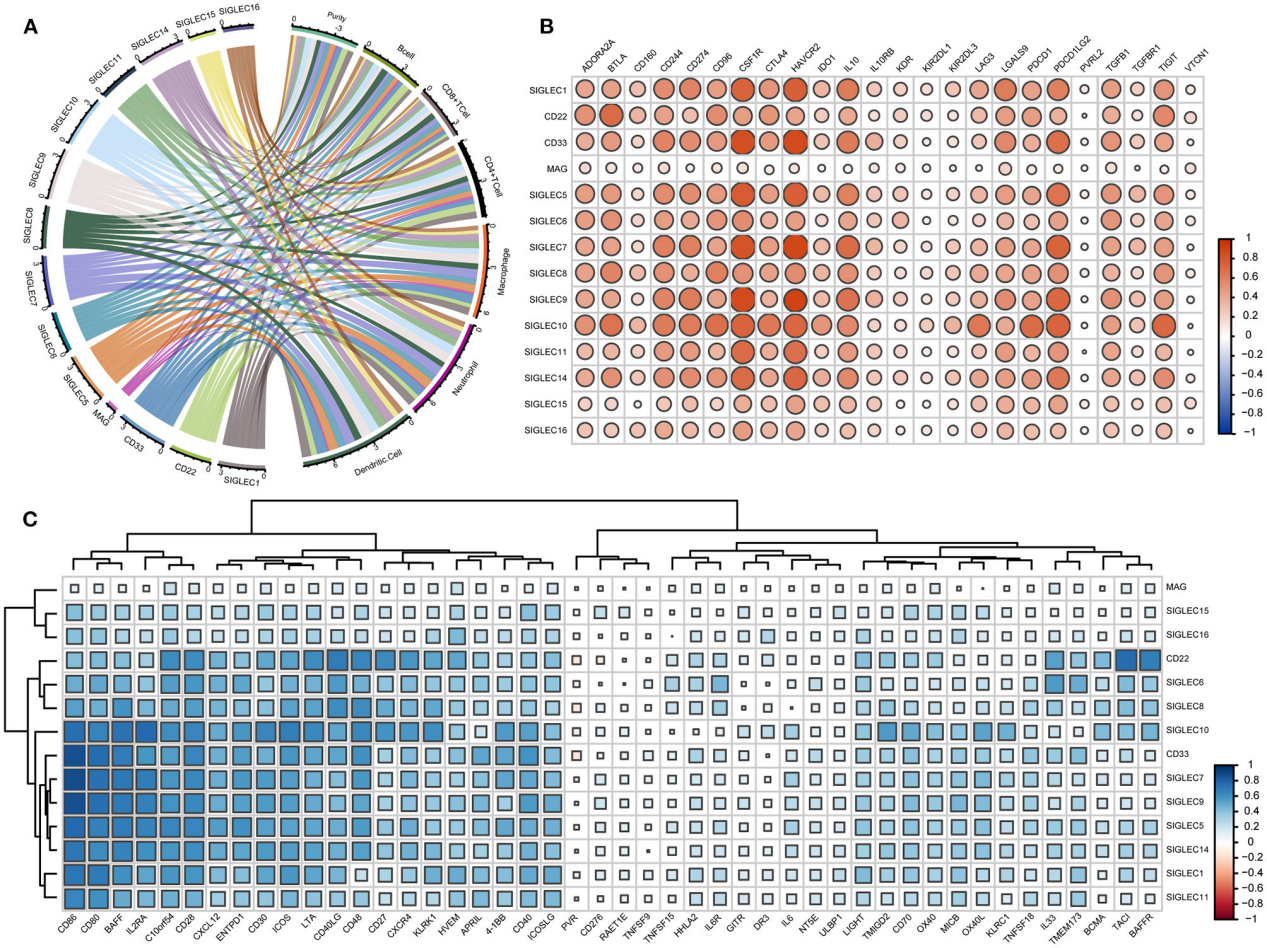
SIGLECs expression is highly correlated with immune-cell infiltration and immune-molecule in LUAD. **(A)** Relationship between SIGLECs and immune-cell infiltration in LUAD. Line thickness and the size of the area corresponding to SIGLECs and immune cells represent the size of the Spearman's rho value. **(B)** Correlations between SIGLECs and immunosuppressive molecules. **(C)** Correlations between SIGLECs and immunstimulatory molecules. The color and size of the squares represent the *R*-value of Spearman's correlation. Correlation coefficients were classified as weak (0.1 ≤ *r* < 0.3), moderate (0.3 ≤ *r* < 0.5), or strong (*r* ≥ 0.5).

Given that the enrichment-analysis results indicated SIGLEC involvement with various immune-activation and -regulation processes, we evaluated correlations between the SIGLEC levels and immune-cell infiltration and immune-molecule levels. The results showed that levels of all SIGLEC family members were negatively correlated with tumor purity. SIGLEC1, CD22, SIGLEC3, SIGLEC5, SIGLEC6, SIGLEC7, SIGLEC8, SIGLEC9, SIGLEC10, SIGLEC11, and SIGLEC14 displayed similar immune-cell profiles, showing positive correlations with infiltrating levels of B cells, CD8^+^ T cells, CD4^+^ T cells, macrophages, neutrophils, and dendritic cells. Compared with their correlations with B cell, CD8+ T cell, and CD4+ T cell infiltration (*r* > 0.3), SIGLEC1 (CD33), SIGLEC5, SIGLEC7, SIGLEC9, SIGLEC11, and SIGLEC14 showed a higher correlation with macrophage, neutrophil, and dendritic cell infiltration (*r* > 0.5). However, no correlation was found between MAG and these immune cells. Additionally, SIGLEC15 and SIGLEC16 were significantly correlated with neutrophil and dendritic cell infiltration (*p* < 0.05), and a strong positive correlation was observed between CD22 and B cell and CD4^+^ T cell infiltration (Figure 6A).

To further investigate immune-related activity associated with SIGLECs *in vivo*, we analyzed correlations between SIGLEC family members with immunosuppressive molecules, such as adenosine A2a receptor (ADORA2A), CD96, indoleamine 2,3-dioxygenase 1 (IDO1), CTLA4, programmed cell death 1 (PDCD1), lymphocyte-activation gene 3 (LAG3), T cell immunoreceptor with Ig and ITIM domains (TIGIT), transforming growth factor-β receptor 1 (TGFBR1), and immunostimulatory molecules, such as CD27, CD276, CD48, CD70, killer cell lectin-like receptor C1, MHC class I polypeptide-related sequence B, IL-6, 5' nucleotidase ecto (NT5E), poliovirus receptor precursor (PVR), glucocorticoid-induced TNF receptor (GITR), and OX40. In LUAD, the immunosuppressive molecules CD244, CD274, colony stimulating factor 1 receptor (CSF1R), hepatitis A virus cellular receptor 2 (HAVCR2), IL-10, PDCD1 ligand 2 (PDCD1LG2) showed a strong positive correlation with SIGLEC1, CD33, SIGLEC7, SIGLEC8, SIGLEC9, SIGLEC10, and SIGLEC14, and CD22 was strongly positively correlated with ADORA2A, B- and T-lymphocyte attenuator (BTLA), CD96, CTLA4, and TIGIT (*r* >0.5). Additionally, SIGLEC11 was strongly positively correlated with CSF1R and HAVCR2, and there was a significant strongly positive correlation between SIGLEC10 and ADORA2A, BTLA, CD96, IDO1, LAG3, PDCD1, PDCD1LG2, and TIGIT (*p* < 0.05, *r* > 0.5). By contrast, SIGLEC family members showed a weak association with CD160, IL-10RB, kinase insert domain receptor, killer cell immunoglobulin-like receptor two Ig domains and long cytoplasmic tail (KIR2DL) 1, KIR2DL3, PVR ligand 2, TGFBR1, and V-set domain-containing T cell activation inhibitor 1, whereas MAG had no association with any immunosuppressive molecule (Figure 6B).

Analysis of associations with immune-stimulating molecules revealed no correlation between SIGLECs and CD276, HERV-H LTR-associating 2, IL-6, NT5E, PVR, retinoic acid early transcript 1E, GITR, death receptor 3, TNF superfamily member (TNFSF)15, TNFSF9, UL16-binding protein 1 (ULBP1) (Figure 6C). However, a moderate-to-strong positive correlation was found between V-domain Ig suppressor of T cell activation (VISTA), CD28, CD40, CD80, CD86, C–X–C chemokine (CXC) ligand 12, CXC receptor 4, ectonucleoside triphosphate diphosphohydrolase 1, inducible T cell co-stimulator (ICOS), IL2 receptor-α, ICOS ligand, lymphotoxin α, CD30, 4-1BB, and B cell-activating factor (BAFF) with CD22, CD33, and SIGLEC1/5/6/7/8/9/10/11/14. IL33 was strongly correlated with CD22 and SIGLEC6. Notably, that there was also a strong correlation between CD22 and CD27, transmembrane activator and CAML interactor, BAFF receptor.

## Discussion

SIGLEC family members play significant and diverse roles in autoimmune diseases and tumor progression (42, 43), making SIGLECs and sialoglycans attractive targets for anticancer immunotherapies (44). Compared with healthy cells, cancer cells sport a higher density of glycan structures terminating in sialic acid, the ligand for SIGLECs. Tumor cells can engage the sialoglycan-Siglec axis to evade immune control. Interference with sialoglycan synthesis or sialoglycan–Siglec interactions might enhance antitumor immunity (32, 45). In the present study, we analyzed SIGLEC expression in multiple tumors relative to normal tissues and comprehensively analyzed relationships between their differential expression and OS in LUAD patients. The results were consistent with previous reports, specifically that SIGLEC15 is upregulated in various tumors, including bladder urothelial carcinoma, cervical squamous cell carcinoma and endocervical adenocarcinoma, CHOL, colon adenocarcinoma, LUAD, KICH, KIRP, thyroid carcinoma, and uterine corpus endometrial carcinoma to varying degrees and can be used as a novel immune checkpoint for tumor immunotherapy (46). One study performed immunohistochemistry to evaluate the expression and prognostic value of SIGLEC15 in a cohort of 103 LUAD specimens. The results showed that patients with positive SIGLEC15 expression had an unfavorable progression free Survival. There was no significant difference in OS between SIGLEC15 positive and negative patients. In TCGA data, it was found that upregulated SIGLEC15 expression was associated with longer OS in breast invasive carcinoma, BLCA, THCA, head and neck squamous cell carcinoma and UCEC and with longer relapse-free survival (RFS) in liver hepatocellular carcinoma, ovarian serous cystadenocarcinoma, BRCA, and UCEC. By contrast, up-regulated SIGLEC15 expression was associated with shorter OS in kidney renal clear cell carcinoma, pancreatic adenocarcinoma and Sarcoma, and with shorter RFS in SARC and PAAD (47). In a recent article, researchers showed for the first time the *in situ* expression of SIGLEC6 by mast cell (MC) in human colorectal cancer (CRC) tissues. SIGLEC6 is up-regulated on MC when stimulated with hypoxia or colon cancer cells. However, SIGLEC6 expression was not detected in colon cell lines. These findings supported that SIGLEC6 can modulate MC activity in the CRC tumor microenvironment (48). As an inhibitory receptor, SIGLEC6 plays a major role in both the proliferation and effector functions of tissue-like memory B cells (49). SIGLEC6 is expressed on plasmacytoid dendritic cell leukemia, but not on cutaneous granulocytic monocytic leukemia (50). In addition, compared with normal lymphoid cells and some other lymphoid malignancies, SIGLEC6 is highly expressed in mucosa-associated lymphoid tissue lymphoma (51). Tuscano (52) previously characterized the expression of CD22 in lung cancer cells and patient samples, identifying CD22 as a target for therapeutic intervention in lung cancer. However, recent studies repeating this experiment found that CD22 was not expressed at measurable levels on the surface of lung cancer cells, and that tumor cells could not be killed by anti-CD22 immunotoxins (53). In the present study, data based on TCGA database, we showed that CD22 was downregulated in LUAD tissue relative to normal tissue, with the same phenomenon reported in lung squamous cell carcinoma.

Downregulation of SIGLEC1, CD22, CD33, MAG, SIGLEC5, SIGLEC6, SIGLEC7, SIGLEC8, SIGLEC11, and SIGLEC16 in LUAD was not only associated with poor OS but also related to the immune response and cell adhesion according to GO and KEGG analyses. As is known, the immune function of the host is closely related to tumor progression. Immune evasion by cancer cells can promote tumor progression. Cell-adhesion molecules play an important role in tumor invasion and metastasis, both of which are related to changes in adhesion-molecule expression (54). Reduced expression of certain adhesion molecules in tumor cells can weaken adhesion between tumor cells, resulting in their separation from the primary tumor body. Additionally, certain adhesion molecules expressed on tumor cells in blood circulation to adhere to vascular endothelial cells and basement membranes. In the present study, our data indicated that SIGLECs might play roles in LUAD occurrence and development.

Gene mutation is an important factor in biological evolution and cancer. Previous studies show that an R122C substitution in human SIGLEC12 results in an inability of proteins to bind sialic acids (55). Additionally, the SIGLEC10 expressed by tumor-associated macrophages can interact with CD24 to promote immune evasion (56). Additionally, SIGLEC14 expressed in a monocytic cell line interacts with DAP12 to enhance lipopolysaccharide-induced TNF-α secretion, and gene fusion *SIGLEC5* and *SIGLEC14* results in functional deletion of SIGLEC14 (57). The loss of SIGLEC14 caused by SIGLEC14-null allele homozygosity is related to a reduced risk of chronic obstructive pulmonary disease exacerbation (58). CD33 single nucleotide polymorphisms (SNPs) have been implicated in the risk of Alzheimer's disease (AD) and the therapeutic efficacy of acute myeloid leukemia (AML) (59). The C> T allele of rs12459419 leads to an Ala (codon GCC) to Val (codon GTC) amino acid change. The Ala14Val change is present in the signal peptide (amino acids 1-17), which is likely to lead to a decrease in CD33 expression and thus affect the dose and efficacy of GO therapy in patients with AML (60, 61). The CD33 polymorphism rs3865444 and its functional proxy rs124549419 are associated with exon 2 splicing efficiency. The shorter CD33-isoform (D2-CD33), generated as a result of alternate splicing, lacking the ligand binding domain represents a gain of function variant that reduces Alzheimer's disease risk (62). In the present study, we found that *SIGLEC* mutations were the most common alteration in LUAD. Whether these mutations will change the protein structure and cause corresponding functional enhancement or impairment of function remains to be elucidated.

Immune-cell infiltration into tumor tissues, as well as their specific type, distribution, and tissue localization, is of particular importance for tumor development and prognosis (63). In the LUAD TME, tumor-infiltrating immune cells are double-edged sword in the development of lung cancer. On the one hand, they attack and kill cancer cells to inhibit tumor progression; however, they also screen tumor cells that are better-suited for survival in an immunoreactive host, change the TME, or are assimilated by lung cancer cells, ultimately promoting tumor progression (64). Recent studies suggest that CD169^+^ macrophages in tumor regional lymph nodes are positively associated with favorable prognosis in patients with colorectal cancer, bladder cancer, endometrial carcinoma, or malignant melanoma (65–68). SIGLECs show distinct expression patterns on different cell types. Human SIGLECs are predominantly expressed on the surface of immune cells, and most immune cells of the tumor microenvironment are co-regulated by the action of SIGLECs. SIGLEC1 is mainly expressed on macrophages and dendritic cells. CD22 is expressed on B cells, conventional dendritic cells and mast cells. CD33 has a more diverse expression as CD33 is present on myeloid progenitors, monocytes, macrophages, microglia and granulocytes. MAG is expressed only on oligodendrocytes and schwann cells. SIGLEC5 is myeloid-specific, but unlike CD33, it is expressed on neutrophils, monocytes, B cells and mast cells. SIGLEC6, a leptin-binding modulator, is expressed on B cells, monocytes and placental trophoblasts. SIGLEC7 is expressed on natural killer cells, monocytes, mast cells and T cells. SIGLEC8 is found only on “allergic cells” (basophils, mast cells, eosinophils), while SIGLEC9 is expressed on neutrophils, monocytes, natural killer cells, conventional dendritic cells and T cells. SIGLEC10 is present on some B cells, monocytes and eosinophils. SIGLEC11 appears to be restricted to macrophages and microglia. SIGLEC14 is expressed on neutrophils and monocytes, and SIGLEC15 is expressed on osteoclasts, macrophages and dendritic cells. SIGLEC16 is present on macrophages and microglia (19, 26, 69). In the present study, we found that CD22, CD33, and SIGLEC1/5/6/7/8/9/10/11/14/15/16 were significantly negatively associated with tumor purity and significantly positively associated with B cell, CD8^+^ T cells, and CD4^+^ T cell infiltration and moderately (*r* > 0.3)-to-strongly (*r* > 0.5) correlated with neutrophil, macrophage, and dendritic cell infiltration. Dendritic cells are antigen-presenting cells (APCs) and central to initiating, regulating, and maintaining immune responses while also playing an important role in inducing antitumor immune responses (70). Dendritic cell-based tumor vaccines have been tested clinically and achieved positive outcomes (71, 72).

Levels of immune molecules in the TME are closely related to patient receipt of immunotherapy and reactivity following treatment (73). In the present study, we showed correlations between SIGLEC family members and LUAD-related levels of immune molecules, including the immune-stimulatory checkpoint molecules CD27, CD28, OX40, ICOS and the immune-inhibitory checkpoint molecules CTLA4, PD1, ADORA2A, BTLA, LAG3, and VISTA. CD28 is expressed on the surface of T cells and required for co-stimulatory signaling essential for T cell activation, proliferation, and survival, as well as T helper 2 cell development. CD28 binds CD80 and CD86 on the surface of APCs to initiate co-stimulatory signaling to T cells (74). By contrast, CTLA-4 delivers a co-inhibitory signal via CD80/CD86 (75). A recent study reported that SIGLEC9-expressing T cells co-expressed several inhibitory receptors, including T cell Ig- and mucin-domain-containing protein 3, PD-1, and LAG3 (25). ICOS expressed on the surface of activated T cells can enhance all basic T cell responses to a foreign antigen according to binding of its unique ligand is ICOSL, which initiates a pathway that enhances antitumor immune responses. Combining ICOS-agonistic or -antagonistic antibodies with CTLA-4 or PD-1/PD-L1 might produce potent synergistic effects (76). BTLA, an immunomodulatory receptor similar to CTLA-4 and PD-1, binds to herpesvirus-entry mediator to initiate co-suppressive signals and play a negative regulatory role in the antitumor immune response. This molecule is related to tumor immune evasion and might represent a potential target for tumor immunotherapy (77). Despite therapies targeting CTLA4, PD1, and PDL1 have shown success in many cancers, although not all patients respond well to these therapies. TIGIT and CD96 are expressed on the surface of T cells and natural killer cells and represent targets for immune modulation (78), as blocking CD96 or TIGIT with monoclonal antibodies improves tumor control in mice, especially when used in combination with PD-1/PD-L1 blockade (79). IL-33, an activator of T cells, mediates its immune response mainly through polarized T helper 2 cells. A study showed that IL-33 enhances SIGLEC8-induced eosinophil apoptosis (80). Overexpression of CSF1R or its ligand CSF1 is found in various solid tumors, including those associated with breast, prostate, and ovarian cancer, and plays an important role in tumor malignancy and metastasis (81). Moreover, the CSF1/CSF1R pathway is a dominant regulator of macrophage differentiation and function, with studies showing that CSF1R inhibition can deplete tumor associated macrophages and improve T cell responses (82, 83). In the present study, correlations between SIGLECs and immune molecules suggest an underlying novel feature of SIGLEC-driven immune response and regulation of immune infiltrating cells. Remarkably, we found that MAG has no or only a very weak correlation with these immune cells and immune molecules. This result seems reasonable, since MAG, in contrast to other SIGLECs, appears to be solely expressed in myelin oligodendrocytes and Schwann cells but not in human immune cells and does not carry any immunoreceptor tyrosine-based inhibitory motifs (84).

This study had limitations. First, the analyses were conducted using publically available databases, and the sample size of the data was relatively small. Second, the results lack experimental and clinical validation. Third, the precise mechanism by which SIGLECs function in LUAD requires further study. LUAD tumorigenesis results from abnormal expression of many genes. Therefore, we hope that these data promote further exploration of targets affecting LUAD occurrence and development.

In conclusion, this study showed that downregulated expression of SIGLEC1, CD22, CD33, MAG, SIGLEC5, SIGLEC6, SIGLEC7, SIGLEC8, SIGLEC11, SIGLEC14, and SIGLEC16 in LUAD was associated with poor prognosis. Additionally, we found that *SIGLEC* mutations in LUAD account for a high proportion of genetic alterations. Moreover, we demonstrated various correlations between SIGLEC levels and different immune molecules, as well as levels of immune-cell infiltration of the TME, offering novel insights and enhancing our understanding of the potential relationships between the TME and LUAD prognosis. Furthermore, these findings might help explain why abnormal expression of certain SIGLEC family members is related to poor prognosis in LUAD patients and thereby offer a useful reference for personalized immunotherapy and predicting the efficacy of immune-checkpoint blockade.

## Data Availability Statement

The datasets presented in this study can be found in online repositories. The names of the repository/repositories and accession number(s) can be found in the article/supplementary material.

## Author Contributions

QY, SN, and HZ developed the study concept and designed the research. HZ analyzed the data and drafted the manuscript. YX, HY, XX, ZH, YY, and WX revised the manuscript. All authors read and approved the final version.

## Conflict of Interest

The authors declare that the research was conducted in the absence of any commercial or financial relationships that could be construed as a potential conflict of interest.

## Abbreviations

TCGA: The Cancer Genome Atlas
PPI: protein–protein interaction
GO: Gene Ontology
KEGG: Kyoto Encyclopedia of Genes and Genomes
BP: Biological processes
CC: cellular components
MF: molecular function
DAVID, The Database for Annotation: Visualization and Integrated Discovery
TIMER: Tumor Immune Estimation Resource.
